# Effect of physical activity on anthropometric and physiological parameters in preschool and school-aged children: a systematic review and meta-analysis of randomized controlled trials

**DOI:** 10.3389/fpubh.2025.1592098

**Published:** 2025-09-10

**Authors:** Nina Wang, Qinglei Wang, Mengyan Wang, Mohd Nazri Bin Abdul Rahman

**Affiliations:** ^1^Faculty of Liberal Arts and Law, Guangdong University of Petrochemical Technology, Maoming, Guangdong, China; ^2^School of Physical Education, Guangdong University of Petrochemical Technology, Maoming, Guangdong, China; ^3^Department of Educational Psychology and Counselling, Faculty of Education, Universiti Malaya, Kuala Lumpur, Malaysia

**Keywords:** preschool, school-based, children, obesity, exercise, meta-analysis

## Abstract

**Background:**

Early childhood is an essential phase characterized by physical, social, and cognitive development. This developmental stage lays the foundation for establishing lifelong health behavior patterns that can resonate into adolescence and adulthood. Despite the growing recognition of the significance of physical activity during crucial developmental periods, the advent of technology-driven modern society has induced a notable preference among children for a sedentary lifestyle. In addition, compared to research involving older children and adolescents, the existing evidence for preschool and school-based remains relatively limited in scope and depth.

**Objective:**

To assess the influence of exercise interventions on anthropometric factors, encompassing Body Mass Index (BMI), skinfold thickness (ST), BMI z-score, and Waist Circumference (WC), as well as physiological parameters, including Diastolic Blood Pressure (DBP) and Systolic Blood Pressure (SBP), in both preschool and school-based children, and the comparison between these two age groups.

**Methods:**

Four databases were searched (such as Web of Science, The Cochrane library, Scopus, and Embase) and included only randomized controlled trials (RCTs) assessing exercise interventions’ impact on anthropometric measurements and blood pressure in children aged 1–12 years, including both preschoolers and school-aged children. The analysis used the standardized mean difference as the outcome measure and employed a random-effects model for data analysis.

**Results:**

From the results, including 29 RCTs, exercise interventions were linked to favorable reductions: BMI (*μ* = −0.317; 95% CI: −0.570 to −0.064), WC (*μ* = −0.010; 95% CI: −0.104–0.085), and ST (*μ* = −0.066; 95% CI: −0.293–0.161). Also, improvements occurred in DBP (*μ* = −0.068; 95% CI: −0.139–0.002) and SBP (*μ* = −0.186; 95% CI: −0.373–0.001). Subgroup analysis found no significant age group differences.

**Conclusion:**

This meta-analytical method provides substantial evidence affirming the effectiveness of physical exercise programs, regardless of age group, with a focus on gross motor skills, whether implemented alone or in conjunction with supplementary interventions, in reducing anthropometric parameters.

**Systematic Review Registration:**

PROSPERO 2023 (CRD42023470312).

## 1 Introduction

Early childhood is a critical phase marked by physical, social, and cognitive development (1). This developmental period establishes the foundation for lifelong health behavior patterns that can extend into adolescence and adulthood (2, 3). In recent years, research has increasingly highlighted the importance of regular physical activity among preschool and school-aged children as a cornerstone of healthy growth and development. Participation in physical activity during these formative years not only offers immediate benefits but also holds potential for long-term improvements in both physical and psychological wellbeing (4, 5). Despite increasing recognition of the importance of physical activity during key developmental stages, the rise of technology-driven lifestyles has fostered a preference for sedentary behaviors among children (6). Moreover, compared to research on older children and adolescents, the body of evidence focusing on preschool and early school-aged populations remains relatively limited in both scope and depth.

Childhood obesity has become a worrying global public health problem. The overall prevalence of obesity in children and adolescents was 8.5% (95% CI 8.2–8.8). The prevalence varied across countries, ranging from 0.4% (Vanuatu) to 28.4% (Puerto Rico). Higher prevalence of obesity among children and adolescents was reported in countries with Human Development Index scores of 0.8 or greater and high-income countries or regions. Compared to 2000–2011, a 1.5-fold increase in the prevalence of obesity was observed in 2012–2023. The pooled estimates of overweight and excess weight in children and adolescents were 14.8% (95% CI 14.5–15.1) and 22.2% (95% CI 21.6–22.8), respectively (7).

According to the WHO (2021), approximately 39 million children under the age of five are overweight or obese, and more than 340 million children and adolescents aged 5–12 fall into this category. This condition not only increases the risk of metabolic disease in adulthood but also impacts children’s quality of life from an early age. Regular physical activity is a key strategy for obesity prevention. The WHO (2019) recommends that children aged 5–12 engage in at least 60 min of moderate to vigorous physical activity daily. Several studies have shown that physical exercise can improve body composition, increase cardiorespiratory fitness, and reduce cardiometabolic risk factors (8, 9). Underlying mechanisms include increased energy expenditure, fat oxidation, and decreased systemic inflammation.

Several previous systematic reviews have assessed the effectiveness of physical activity interventions in children, but most have focused on specific age groups, such as preschool or school-aged children. There has been no comprehensive meta-analysis directly comparing the effects of interventions in these two age groups, particularly regarding anthropometric indicators (BMI, waist circumference, skinfold thickness) and physiological parameters (systolic and diastolic blood pressure) (8–11). However, differences in physical and cognitive developmental stages between preschool and school-age children may influence response to interventions.

Therefore, this study aimed to systematically and quantitatively evaluate the effects of physical activity interventions on anthropometric and physiological parameters in preschool children (1–5.99 years) and school-age children (6–12 years), and to compare the effectiveness of interventions in these two age groups.

## 2 Methods

### 2.1 Registration and protocol guidelines

This study adhered to the Preferred Reporting Items for Systematic Reviews and Meta-Analyses (PRISMA) guidelines (12).

### 2.2 Source of data

A comprehensive search was conducted using relevant keywords across multiple databases, including Web of Science, The Cochrane Library, Scopus, and Embase, to identify pertinent studies. The search syntax is detailed in the supplementary file.

### 2.3 Inclusion criteria and study selection

Eligible studies focused on children aged 1–5.99 (preschool) and 6–12 years (school-aged), of both genders. Participants were required to be overweight or obese but without any diagnosed medical conditions. Only randomized controlled trials (RCTs) that assessed at least one anthropometric or physiological parameter (i.e., DBP, SBP, BMI, ST, WC, BMI z-score) were included.

Exclusion criteria encompassed studies involving children with clinical conditions such as diabetes or hypertension; studies that reported only lifestyle changes without structured physical activity; studies including participants over age 12; and studies lacking a control group. Two authors independently screened titles, abstracts, and full texts. Disagreements were resolved through consultation with a third reviewer. Only studies published in English were considered.

### 2.4 Extraction of data

Two reviewers assessed titles and abstracts of the identified records. Full texts of potentially eligible studies were then examined in detail. Data were extracted using a standardized template covering study design, sample size, participant demographics (age, gender), intervention details (type, duration), and outcome measures.

### 2.5 Quality appraisal of included studies

The Physiotherapy Evidence Database (PEDro) tool was used to evaluate trial quality, considering factors such as study design, sample size, blinding procedures, and allocation concealment (13).

### 2.6 Synthesis of data

Pooled effect sizes were calculated to assess the relationship between exercise interventions and health outcomes. Subgroup analyses explored potential sources of heterogeneity, such as participant characteristics and specific intervention features.

### 2.7 Statistical analysis

A random-effects model using standardized mean differences was applied. Heterogeneity was estimated via τ^2^ using a restricted maximum-likelihood estimator (14), and further assessed using the Q-test for heterogeneity (15) and the I^2^ statistic (16). In the presence of heterogeneity (τ^2^ > 0), prediction intervals were calculated (17). Studentized residuals and Cook’s distances identified outliers and influential studies (18). Outliers were defined as studies with studentized residuals exceeding the 100 × [1–0.05/(2 × k)]th percentile, accounting for a Bonferroni correction. Influential studies had Cook’s distances exceeding the median plus six times the interquartile range. To detect funnel plot asymmetry, both the rank correlation test (19) and regression test (20), were employed, using the standard error of observed outcomes as a predictor. Analyses were performed in R (version 4.3.1) (R Core Team, 2020) using the meta package (version 4.2.0) (21).

### 2.8 Publication bias

To assess funnel plot asymmetry, the researcher utilized the rank correlation test proposed by Begg and Mazumdar (19) and the regression test developed by Sterne and Egger (19, 20).

### 2.9 Strength of evidence

The robustness of evidence was evaluated using the GRADE framework, which considers study design, risk of bias, consistency, precision, and directness of evidence.

## 3 Results

### 3.1 Process of study selection

**Figure 1:**
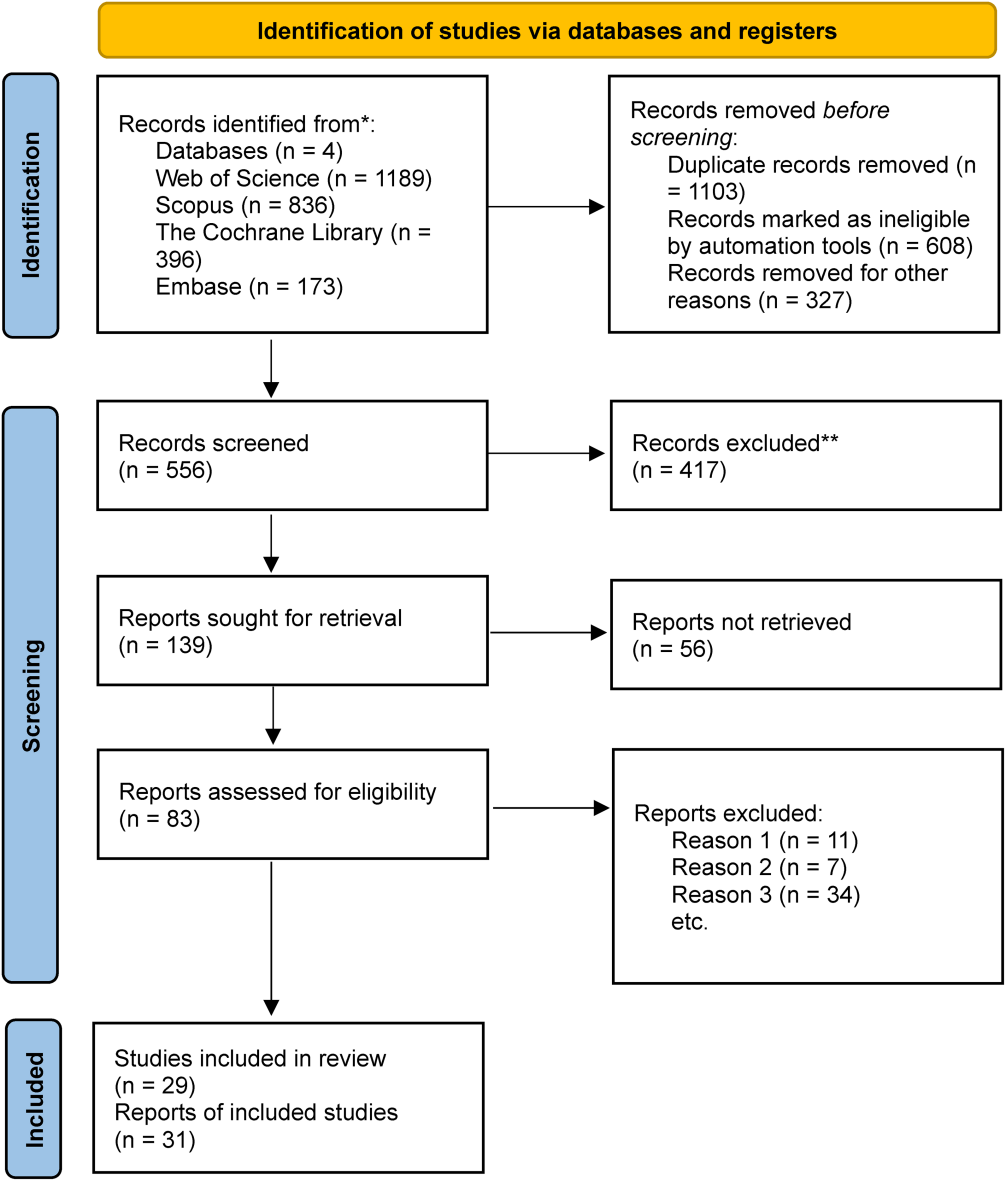
PRISMA flow diagram.

The electronic search yielded 2,594 records. After removing duplicates and screening titles and abstracts, 2035 articles were excluded. From the remaining 556, full-text screening was conducted for 83 studies. Reference lists of included studies and relevant reviews were also examined for additional sources. Full-text exclusions were due to improper study design (11 studies), ineligible populations (7 studies), or inappropriate outcome measures (34 studies). Ultimately, 31 studies were included in the systematic review, and 29 met the criteria for meta-analysis (22–52). The PRISMA flowchart is shown in Figure 1 (53).

### 3.2 Study characteristics

Table 1 summarizes the characteristics of the included studies. The final analysis encompassed 5,260 preschoolers and 3,929 school-aged children. Participants came from various countries, including Spain (*n* = 6), USA (*n* = 4), Germany (*n* = 4), Australia (*n* = 3), Italy (*n* = 2), China (*n* = 2), Norway (*n* = 1), Berlin (*n* = 1), Portugal (*n* = 1), Netherlands (*n* = 1), Saudi Arabia (*n* = 1), Greece (*n* = 1), Israel (*n* = 1), Switzerland (*n* = 1), and the United Kingdom (*n* = 1). All studies included both boys and girls. Sample sizes varied, from 27 participants (36) to 1,154 (23). Intervention durations ranged from 5 weeks (36) to 96 weeks (33).

**Table 1:** Characteristics and findings of the studies.

First author (year), country	Participants	Intervention	Frequency/(days in week)/time (mins)	Outcomes	PEDro score
Espinoza Silva et al. (28), Spain	443 (NA), (6.37 *±* 0.65 y); IG (*n* = 295), CG (*n* = 149)	HIIT training	28-week, (2.t.w), (40–50 min)	↓BMI, ↓WC, ↓Body fat, ↔SBP, ↔DBP, ↓ST, ↔cardiometabolic risk	7
Migueles et al. (39), Spain	92 (39% girls); (8-11y); IG (*n* = 47), CG (*n* = 45)	Aerobic + resistance training	20-week, (3-5.t.w), (90 min)	↔BMI, ↔BMI z-score ↔WC, ↓SBP, ↓DBP, ↔Fat mass, ↔CRF, ↓LDL, ↓HDL, ↓Triglycerides	8
Vandoni et al. (50), Italy	37 (22.5%); (11 ± 1.9 y); No control	Online aerobic Interval Training + Muscular strength	12-week, (3.t.w), (60 min)	↔BMI, ↓BMI z-score, ↓WC, ↔SBJ, ↔Walking, ↑PA, ↑Physical fitness	5
Stavnsbo et al. (46), Norway	1,129 (NA); (10.2 ± 0.3 y); IG (*n* = 596), CG (*n* = 533)	PA program	28-week, (5.t.w), (60 min)	↔BMI, ↔WC, ↔CRF, ↔DBP, ↓HDL, ↔CRF, ↔ LDL, ↑MVPA, ↔SBP, ↓Triglyceride	9
Ketelhut et al. (34), Berlin	105 (51% girls); (8.2 ± 0.6 y); IG (*n* = 51), CG (*n* = 50)	Running + ball game, relay + motor skills	37-week, (5.t.w), (45 min)	↔BMI, ↓DBP, ↔SBP	7
Brasil et al. (26), Portugal	35 (48.57%); (11.1 ± 1.1 y); IG (*n* = 20), CG (*n* = 15)	Judo training	12-week, (2.t.w), (60 min)	↓BMI, ↓ BMI z-score, ↓WC, ↔ HR, ↓ SBP, ↔DBP, ↓% Fat (%), ↔AMM, ↔VO_2_ max	7
Aguilar-Cordero et al. (22), Spain	98 (NA); (10.65 y); IG (*n* = 49), CG (*n* = 49)	Games and sports appropriate to their capacities: aerobic and jumping	36-week, (4.t.w), (90 min)	↓SBP, ↓DBP, ↔Fat (%)	8
van Leeuwen et al. (49), Netherlands	254 (57.1% girl); (6–12 y); IG (*n* = 125), CG (*n* = 129) Not control	Kids4Fit program	12-week, (2.t.w), (60 min)	↔SBP, ↓DBP, ↔Suttle-run test score	4
Mannarino et al. (37), Italy	27 (33.3% girls), (11 ± 2 y) No control	Playful and recreative activities	12-week, (3.i.w), (60 min)	↔BMI, ↔ BMI z-score, ↔ WC, ↓SBP, ↔DBP	5
Nambi et al. (40), Saudi Arabia	76 (NA); (5–12 y); IG (*n* = 38), CG (*n* = 38)	High-intensity aerobic training + diet	8 weeks, (4.t.w), (50 min)	↔BMI, ↔Adiponectin, ↔Leptin, ↔TNF-*α*, ↔IL-6	9
Martínez-Vizcaíno et al. (38), Spain	487 (52.15% girls); (4–6 y) IG (*n* = 248), CG (*n* = 239)	The MOVI-Kids program: sports games + playground games + dance + motor skills	36-week, (3.t.w), (60 min)	↔BMI, ↑CRF, ↔DBP, ↔MAP ↔SBP, ↑ Motor skills, HDL, LDL, Glucose, insulin	6
Lima et al. (36), Australia	499 (52.05% girls), (6–12 y); IG (*n* = 293), CG (*n* = 206)	Aerobic + Strength _ + Motor skill tasks	5-week, (5.t.w), (45 min), Two other days (10 min)	↓ST, ↑Aerobic fitness, ↔Insulin, ↓Triglycerides, ↓Cholesterol, ↓HDL, ↔Glucose	9
Genitsaridi et al. (30), Greece	705 (52% girls); (10.09 ± 2.86 y) IG (*n* = 579) CG (*n* = 126)	Physical activity program: walking, jogging, dancing or bicycling	48-week, (6-7.t.w), (30–45 min)	↓BMI, ↓WC, ↓DBP, ↓SBP	6
Williams et al. (51), USA	175 (61% girls), (8–11-y), IG (*n* = 90), CG (*n* = 85)	Aerobic exercise: Vigorous aerobic activities + running games +ball games+ jump rope	32-week, (5.t.w), (60 min)	↓Body fat, ↑VO_2_ peak, ↑Quality of life, ↔Self-worth, ↓Depression ↑Anger control, ↔Global self-worth	8
Hacke et al. (31), Germany	135 (52.5% girls); (4.8 ± 0.8 y), IG (*n* = 92), CG (*n* = 43)	Psychomotor forms of play and dances + functional gymnastics	24-week, (2.t.w), (45 min)	↔BMI; ↓DBP; ↓ SBP; ↓ PWV; ↔WC, ↔PA levels; ↔MVPA	6
Latorre-Román et al. (35), Spain	111 (46.4% girls); (4.4 ± 0.6 y); IG (*n* = 56), CG (*n* = 55)	Aerobic games and gross locomotor movement	10-week, (3.t.w), (30 min)	↔BMI, ↔WC, ↑Physical fitness	6
Ketelhut et al. (33), Germany	172 (NA); (3.0 ± 0.4 y); IG (*n* = 90), CG (*n* = 82)	Different joyful games: strength, conditioning, and coordination skills	96-week, (3.t.w), (45 min)	↓DBP, ↓ SBP, ↑Motor skills	6
Tan et al. (57), China	42 (49% girls); (5.1 ± 0.3 y); IG (*n* = 21), CG (*n* = 21)	PA program: quick walking, slow running, jumping, rope skipping, semi-squatting, slow crawling	10-week, (5.t.w), (60 min)	↓BMI, ↓WC, ↓Body, ↓fat mass, ↓SBP, ↔DBP, ↑Physical fitness	5
Serra-Paya et al. (45), Spain	113, (44.1% girls), (6–12 y); IG (*n* = 54), CG (*n* = 59)	PA program	32-week, (3.t.w), (90 min)	↔BMI, ↔WC, ↑MVPA	9
Serra-Paya et al. (45), Spain	709 (49.5% girls), (4.7 ± 0.6 y), IG (*n* = 367); CG (*n* = 337)	Coordinative skills and perception (optical, acoustical, tactile, vestibular, and kinesthetic)	48-week, (5.t.w), (30 min)	↔BMI, ↑ motor skills, ↔SBP, ↔DBP, ↔ST, ↔ MVPA	8
Donath et al. (27), Switzerland	41 (41.5% girls); (4.4 ± 1.1 y); IG (*n* = 22), CG (*n* = 19)	Fundamental movement skills (rolling, kicking, catching, throwing, dribbling)	6-week, (2.t.w), (30 min)	↔BMI, ↑FMS	5
Fitzgibbon et al. (29), USA	147 (50% girls); (2–5 y), IG (*n* = 72), CG (*n* = 74)	Aerobic activity	14-week, (3.t.w), (20 min)	↓BMI; ↓BMI z-score ↑MVPA (min/day)	5
Annesi et al. (23), USA	1,154 (51.2% girls); (4.4 ± 0.5 y); IG (*n* = 690), CG (*n* = 464)	PA program: gross motor skills (e.g., walking, running, jumping)	36-week, (5.t.w), (30 min)	↓BMI, ↑MVPA	5
Bellows et al. (24), USA	201 (45% girls); (3–5 y), IG (*n* = 98), CG (*n* = 103)	Multiple PA: gross motor skill categories: stability (trunk strength) + locomotor (running, hopping, skipping), + manipulation (ball skills)	18-week, (4.t.w), (15–20 min)	↔BMI, ↔ BMI *z-*score, ↑Gross motor skill, ↔ PA	5
Bocca et al. (25), Germany	62 (72.1% girls); (4.6 ± 0.8 y); IG (*n* = 33), CG (*n* = 29)	PA program: ball playing and dancing to music, motor skills activity	16-week, (1.t.w), (60 min)	↓BMI; ↓BMI z-score; ↓WC; ↓Body fat	6
Zask et al. (52), Australia	498 (48.3% girls); (3–6 y); IG (*n* = 335), CG (*n* = 163)	PA program: fundamental movement skill activity	40-week, (2.t.w), (20 min)	↓BMI *z-*score; ↓WC; ↑FMS	6
Jones et al. (32), Australia	97 (NA); (3–5 y); IG (*n* = 52), CG (*n* = 45)	PA program: *Jump Start* movement skill development	20-week, (3.t.w), (20 min)	↔BMI, ↑Movement skill, ↑PA	7
Nemet et al. (41), Israel	725 (45% girls); (5.2 ± 0.2 y); IG (*n* = 349), CG (*n* = 376)	Physical activity program: soccer, dodge ball, running games with attention given to coordination and flexibility skills	24 weeks, (6.t.w), (45 min)	↔BMI; ↓Obesity, ↑Physical fitness	6
Puder et al. (42), Switzerland	652 (NA); (5.1 ± 0.7 y); IG (*n* = 342), CG (*n* = 310)	PA program	48-week, (4.t.w), (45 min)	↔BMI; ↑Motor agility, ↑Body fat, ↑ST, ↑WC, ↑ Physical fitness	8
Tan et al. (48), China	60 (43% girls), (9–10 y); IG (*n* = 30), CG (*n* = 30)	Physical activity (running, jumping, squatting, crawling, and aerobic dance)	8-week, (5.t.w), (50 min)	↓BMI; ↑ST; ↑WC; ↑Cardiovascular; ↑Fitness index, ↓ST ↑Running; ↑Jumping ability	8
Sacher et al. (44), United Kingdom	81 (NA), (8–12 y); IG (*n* = 36), CG (*n* = 45)	A series of land- and water-based multiskilled games	24-week, (2.t.w), (60 min)	↓BMI, BMI z-score, ↓ WC, ↓LBM; ↓Fat mass; ↓Body fat; ↓SBP, ↓ DBP; ↑PA; ↑self-esteem	8

.t.w, times a week; y, years, IG, Intervention group; CG, Control group; WC, Waist circumference; LBM, lean body mass; SBP, Systolic blood pressure; DBP, diastolic blood pressure; PA, physical activity; ST, Skinfold thickness; FMS, fundamental movement skill; MVPA, Moderate-to-vigorous physical activity; PWV, Pulse wave velocity; HDL, high density lipoprotein, CRF, cardiorespiratory fitness, MAP, mean arterial pressure, CSA, muscle-mass-cross-sectional area, LDL, low-density lipoprotein.

Outcome measures included a broad array of indicators: anthropometric (e.g., BMI, WC, ST, BMI z-score, fat mass, body fat percentage), physiological (e.g., cardiorespiratory fitness, blood pressure), physical (e.g., MVPA, gross motor skills), and blood biomarkers (e.g., adiponectin, leptin, insulin, triglycerides, IL-6, TNF-*α*, HDL, LDL). Full details for each RCT are provided in Table 1.

### 3.3 Meta-analysis of BMI outcome

**Figure 2:**
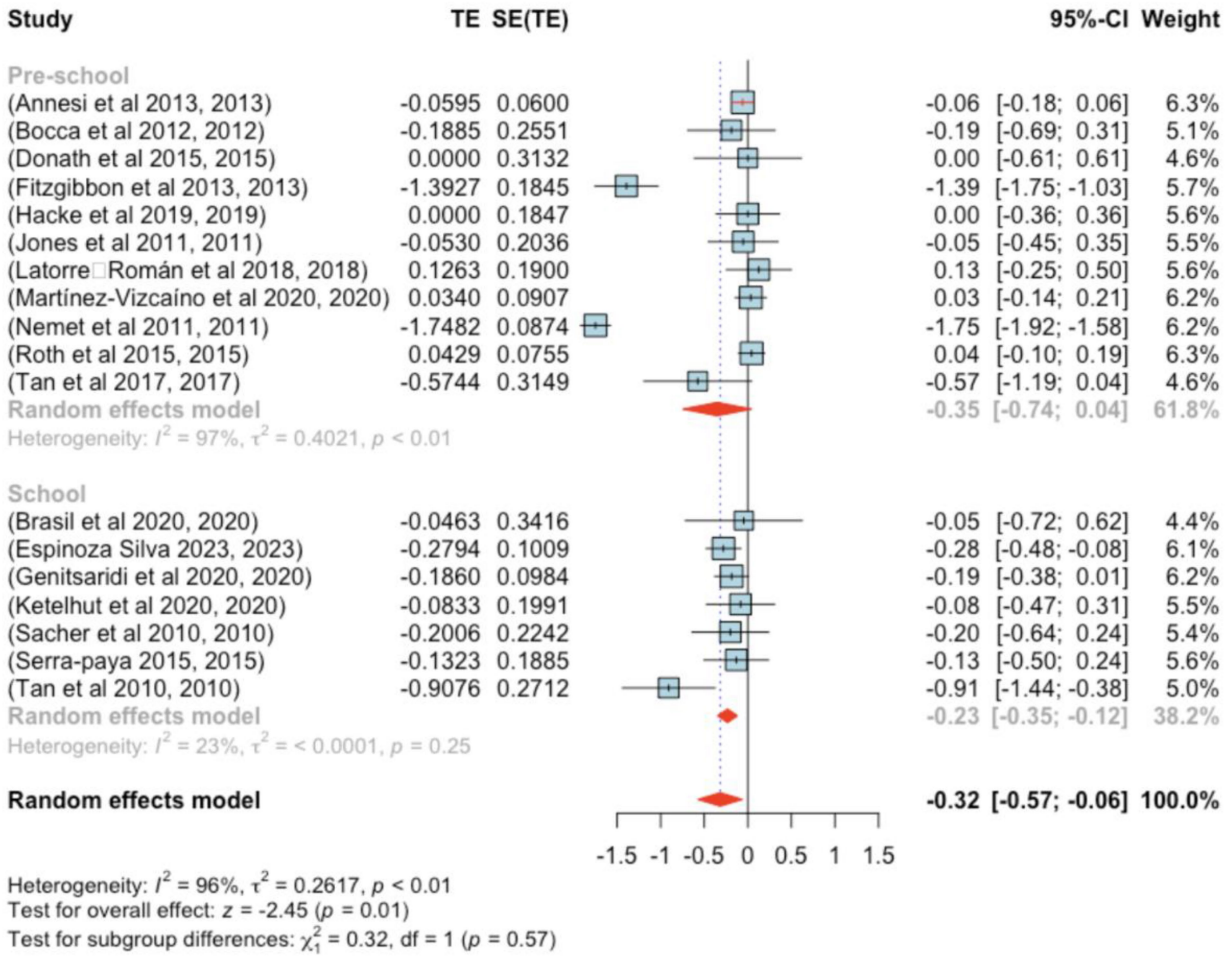
Forest plot showing the observed outcomes and the estimate of the random-effects model.

The analysis incorporated 18 studies. The observed standardized mean differences ranged from −1.748 to 0.126, with most estimates being negative (72%). The estimated average standardized mean difference, based on the random-effects model, was *μ* = −0.317 (95% CI: −0.570 to −0.064). This average outcome significantly differed from zero (*z* = −2.452, *p =* 0.014). Heterogeneity, as indicated by the Q-test, was substantial [Q(17) = 380.228, *p* < 0.001, τ^2^ = 0.262, *I*^2^ = 96.925%]. The 95% prediction interval for true outcomes was −1.351 to −0.717, suggesting the possibility of positive outcomes in some studies. Subgroup analysis between the pre- and school-age groups was not significant (*p =* 0.57) (Figure 2).

One study, Nemet et al. (41), exhibited a studentized residual larger than ±2.991, identifying it as a potential outlier. According to Cook’s distance, two studies (29, 41) were considered overly influential.

**Figure 3:**
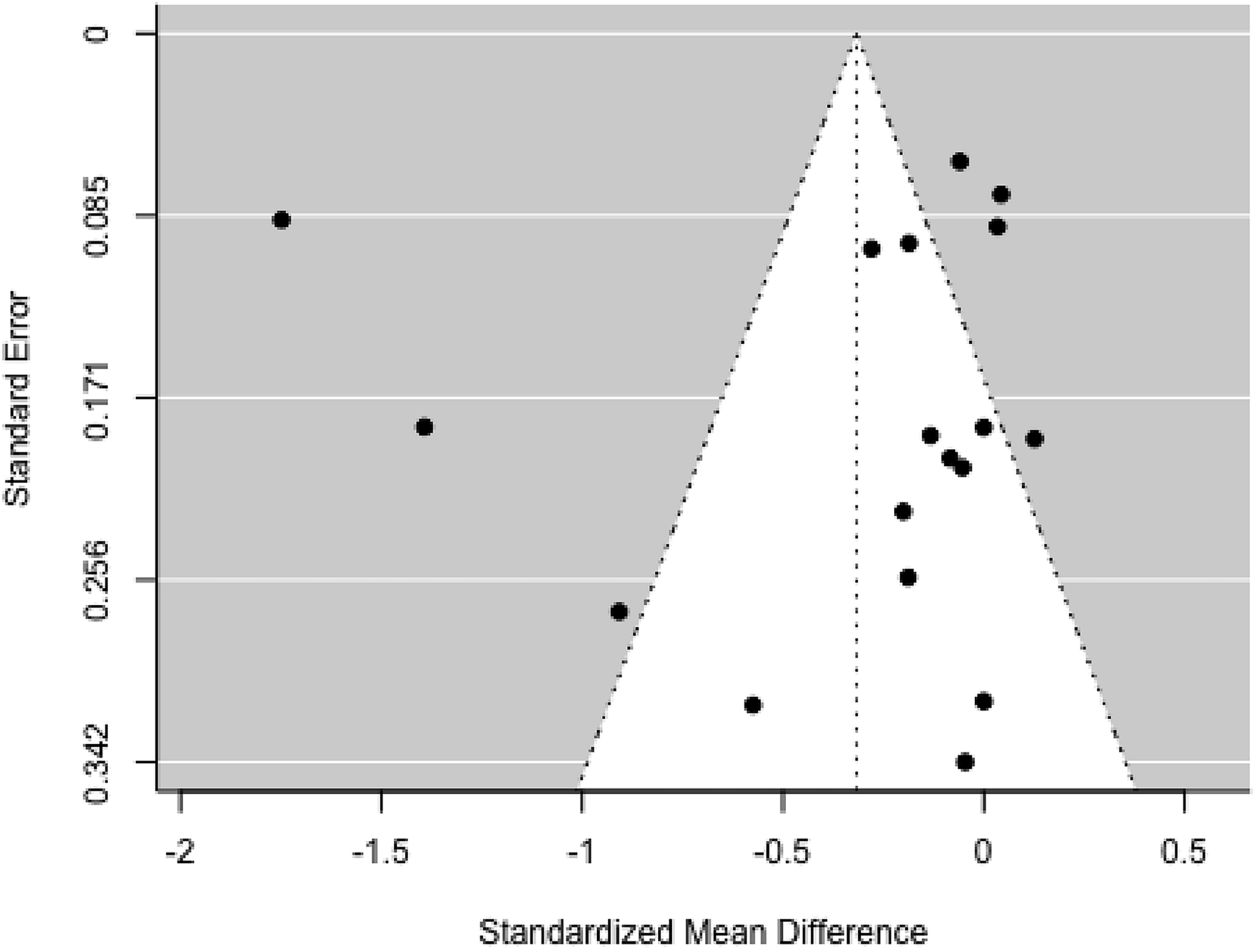
Funnel plot.

Funnel plot analysis (Figure 3) did not indicate significant asymmetry, as confirmed by the rank correlation and regression tests (*p =* 0.068 and *p =* 0.840, respectively).

After excluding potential outlier studies, the estimated average standardized mean difference was *μ* = −0.101 (95% CI: −0.188 to −0.014). This result significantly differed from zero (*z* = −2.273, *p =* 0.023). Although heterogeneity remained, it was no longer significant [Q(15) = 23.199, *p =* 0.080, τ^2^ = 0.008, *I*^2^ = 31.765%]. The 95% prediction interval for true outcomes was −0.300–0.098, indicating the potential for positive outcomes in some studies.

### 3.4 Meta-analysis of BMI Z-score outcome

**Figure 4:**
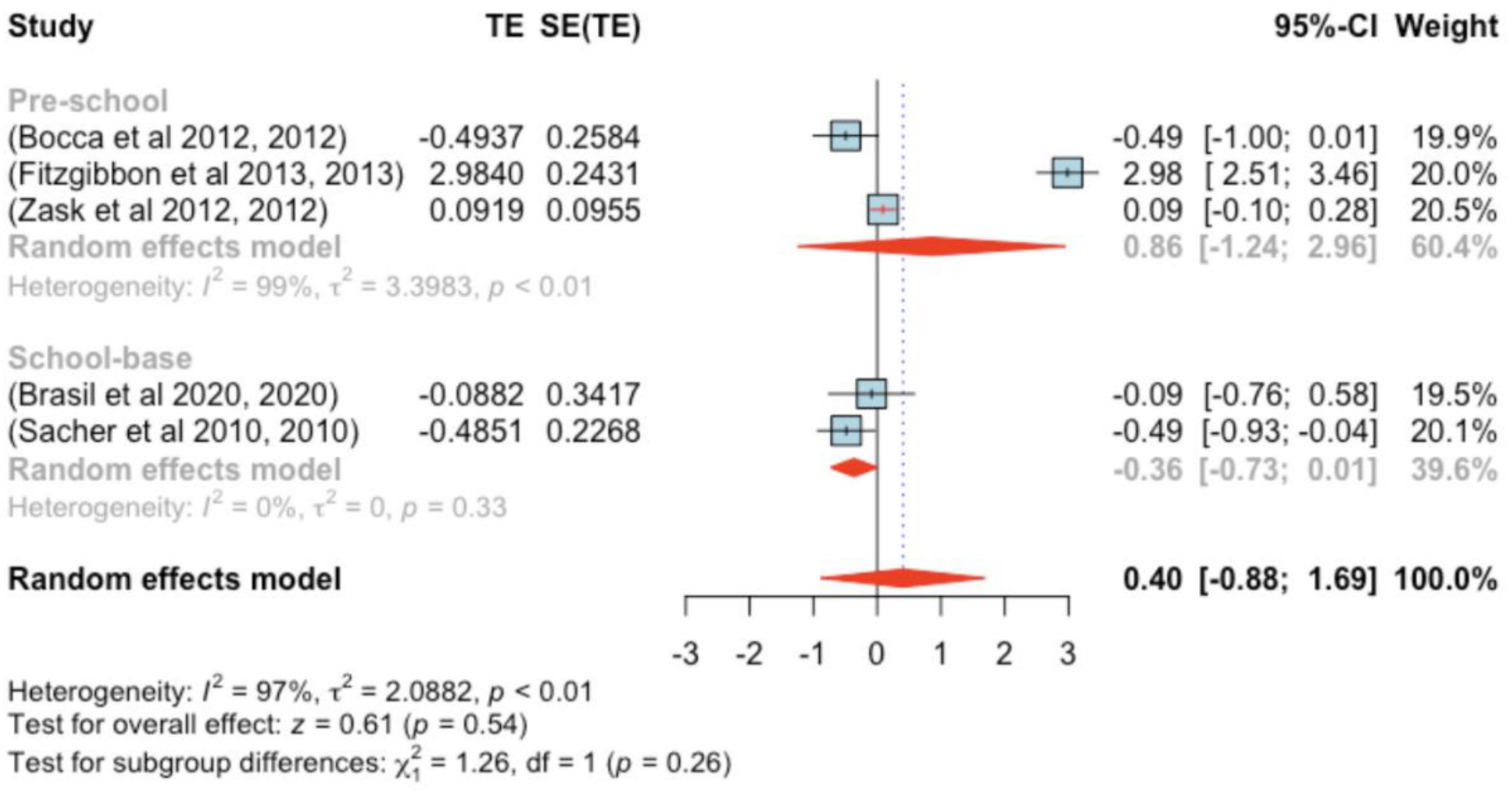
Forest plot showing the observed outcomes and the estimate of the random-effects model.

The analysis included five studies (*k* = 5). Standardized mean differences ranged from −0.494 to 2.984, with the majority being negative (60%). The estimated average standardized mean difference, based on the random-effects model, was *μ* = 0.403 (95% CI: −0.882–1.688). This result did not significantly differ from zero (*z* = 0.615, *p =* 0.539). Substantial heterogeneity was observed [Q(4) = 148.967, *p* < 0.001, τ^2^ = 2.088, *I*^2^ = 97.981%]. The 95% prediction interval for true outcomes ranged from −2.707 to 3.513, indicating the possibility of negative outcomes in some studies. Subgroup analysis between the pre- and school-age groups was not significant (*p =* 0.26) (Figure 4).

One study (29) showed a studentized residual larger than ±2.576, suggesting potential outlier status. Based on Cook’s distances, the same study was deemed overly influential.

**Figure 5:**
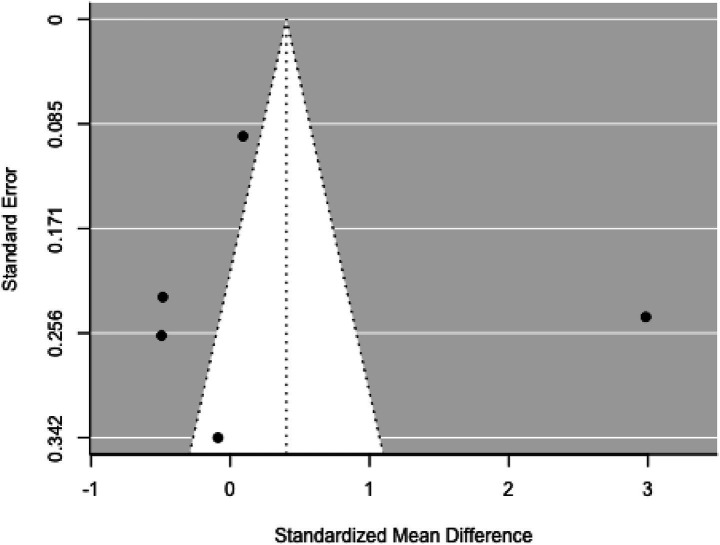
Funnel plot.

Funnel plot assessment (Figure 5) did not reveal significant asymmetry; both the rank correlation and regression tests indicated no evidence of bias (*p =* 0.817 and *p =* 1.000, respectively).

After excluding the outlier study, the estimated average standardized mean difference was *μ* = −0.206 (95% CI: −0.545–0.132), which did not differ significantly from zero (*z* = −1.194, *p =* 0.232).

### 3.5 Meta-analysis of waist circumference outcome

**Figure 6:**
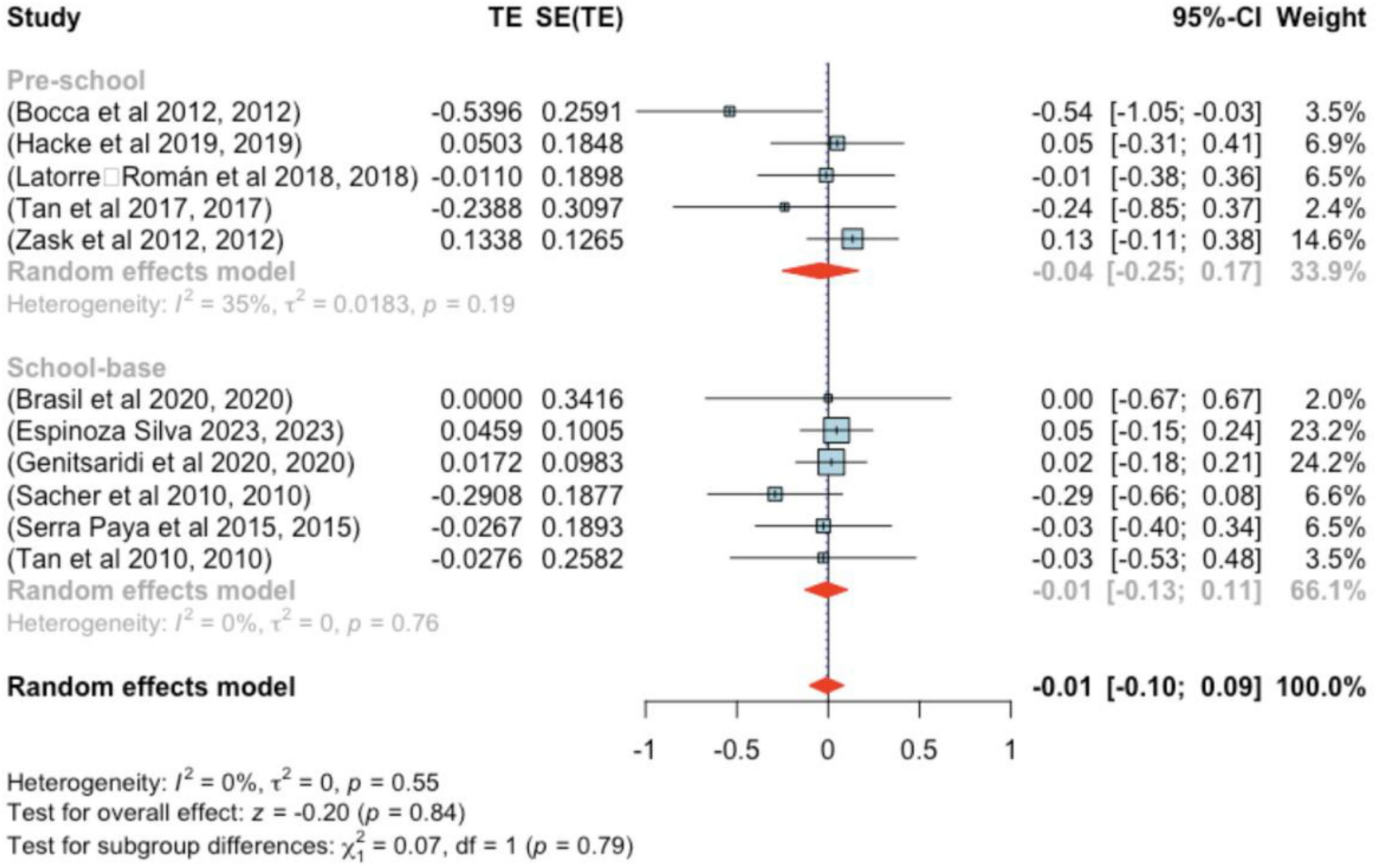
Forest plot showing the observed outcomes and the estimate of the random-effects model.

Eleven studies were included in this segment. Standardized mean differences ranged from −0.540 to 0.134, with most being negative (55%). The estimated average standardized mean difference, based on the random-effects model, was μ = −0.010 (95% CI: −0.104–0.085), with no significant difference from zero (*z* = −0.198, *p* = 0.843). Heterogeneity was not significant [Q(10) = 8.762, *p =* 0.555, τ^2^ = 0.000, *I*^2^ = 0.000%] (Figure 6).

**Figure 7:**
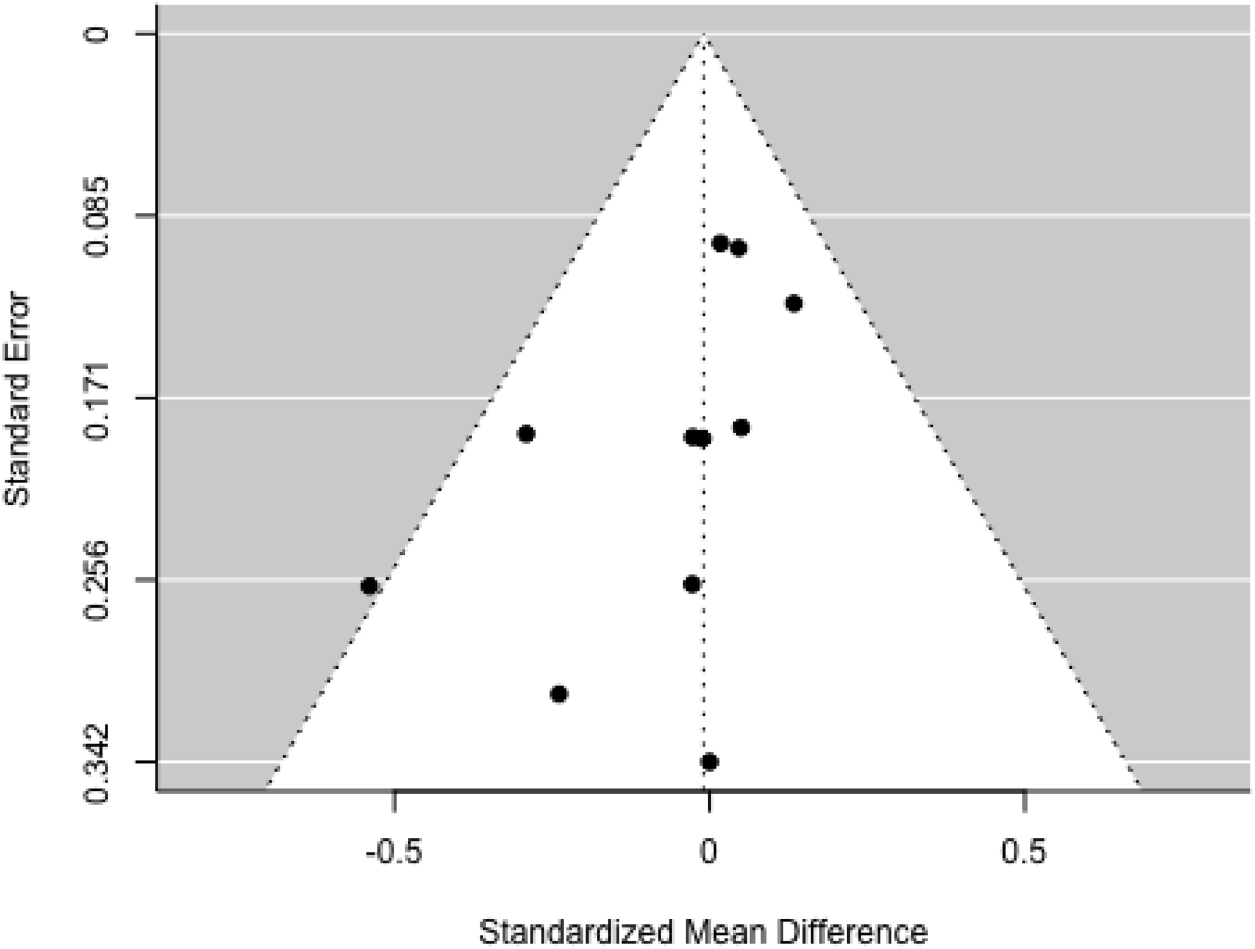
Funnel plot.

None of the studies showed studentized residuals exceeding ±2.838, suggesting no outliers. Cook’s distances indicated that none of the studies were overly influential. Funnel plot analysis (Figure 7) showed no significant asymmetry, as confirmed by the rank correlation and regression tests (*p* = 0.121 and *p* = 0.095, respectively).

### 3.6 Meta-analysis of skin thickness outcome

**Figure 8:**
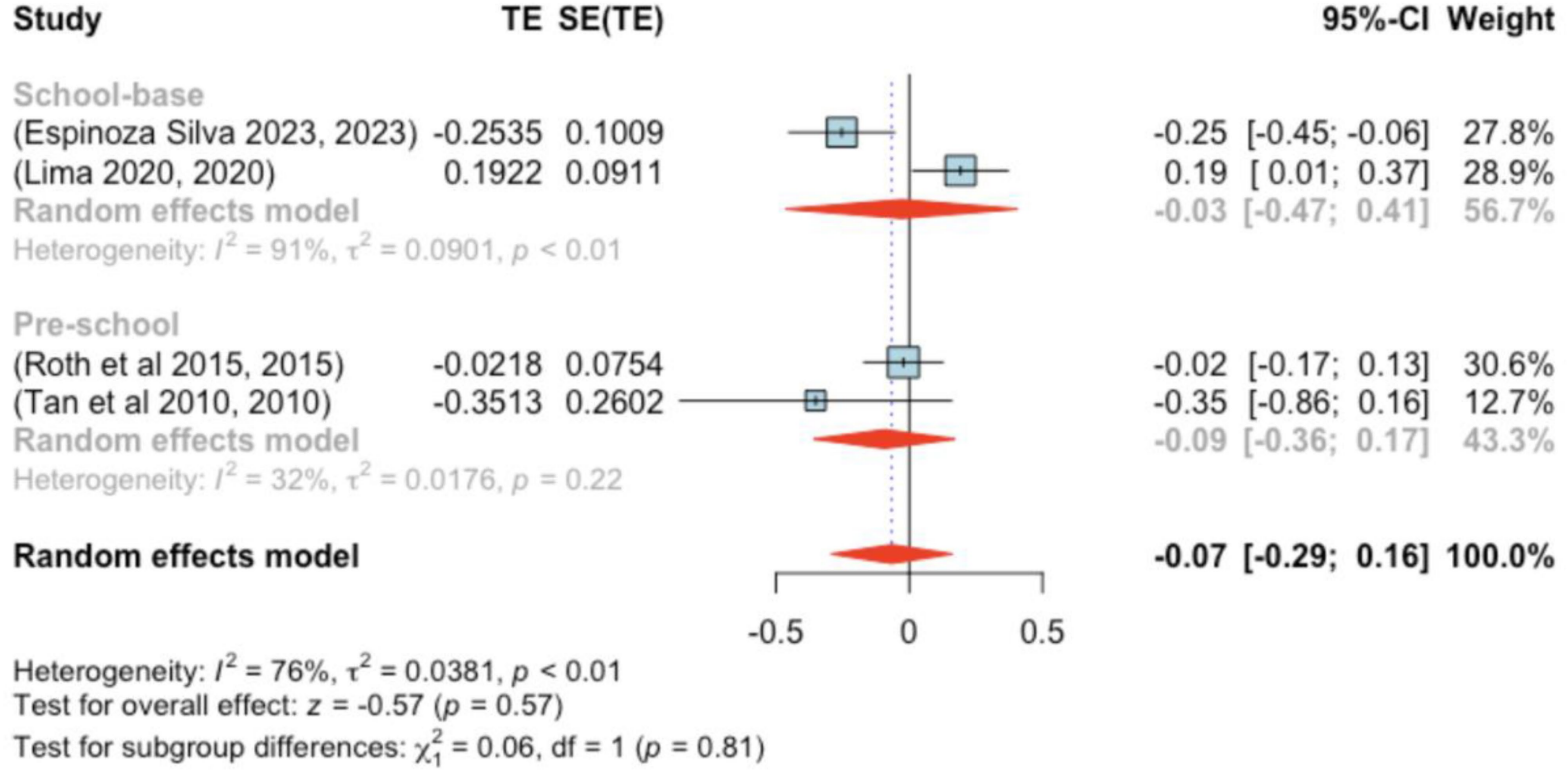
Forest plot showing the observed outcomes and the estimate of the random-effects model.

The analysis included four studies (*k* = 4). Standardized mean differences ranged from −0.351 to 0.192, with the majority being negative (75%). The estimated average standardized mean difference, based on the random-effects model, was *μ* = −0.066 (95% CI: −0.293–0.161). This result did not significantly differ from zero (*z* = −0.571, *p =* 0.568). Heterogeneity was present [Q(3) = 12.386, *p =* 0.006, τ^2^ = 0.038, *I*^2^ = 76.673%]. The 95% prediction interval for true outcomes was −0.511–0.379, suggesting possible positive outcomes in some studies. Subgroup analysis between the pre- and school-age groups was not significant (*p =* 0.81) (Figure 8).

**Figure 9:**
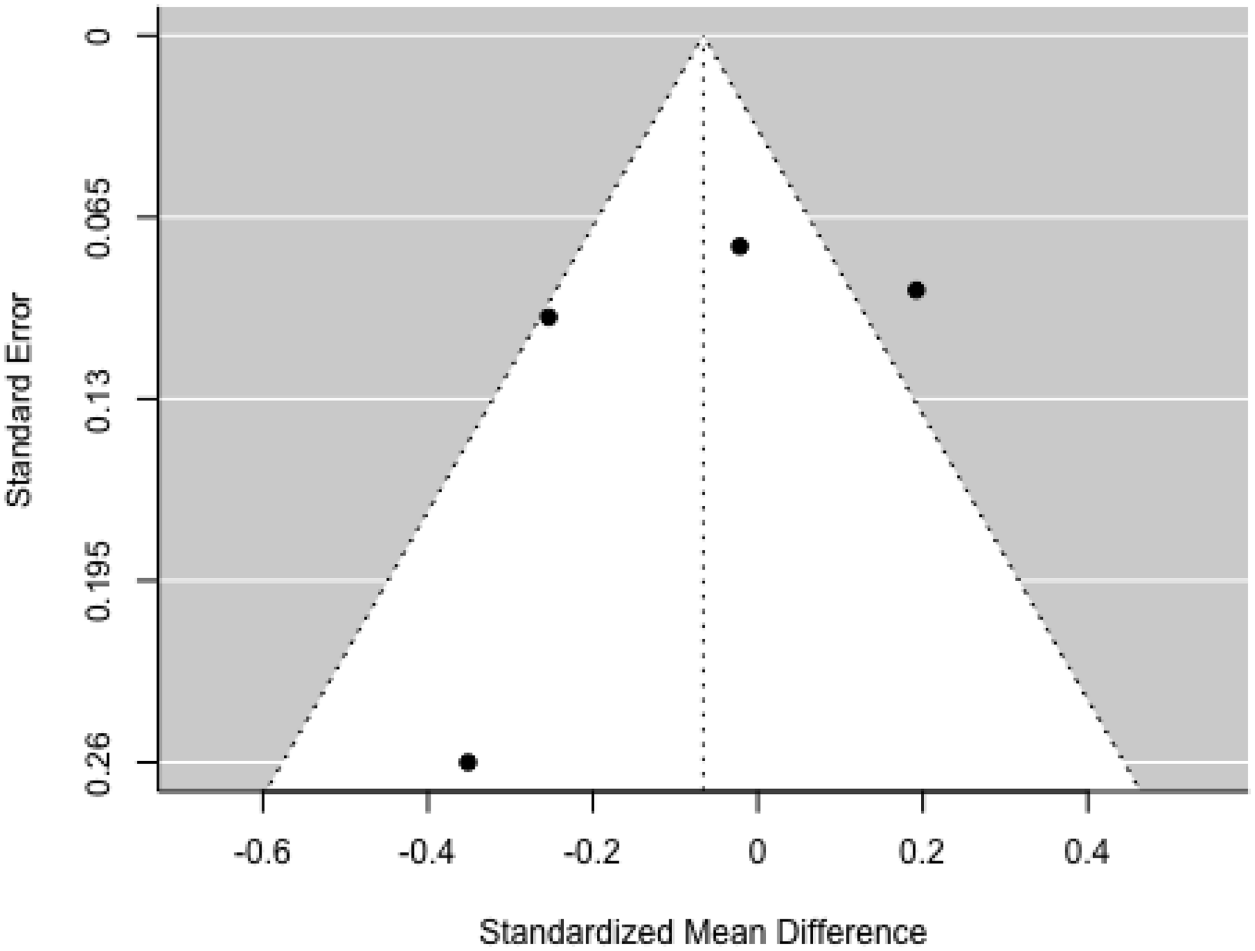
Funnel plot.

None of the studies exhibited studentized residuals beyond ±2.498, and Cook’s distances indicated no overly influential studies. Funnel plot analysis (Figure 9) showed no significant asymmetry, with rank correlation and regression tests also indicating no bias (*p =* 0.750 and *p =* 0.301, respectively).

### 3.7 Meta-analysis of DBP outcome

**Figure 10:**
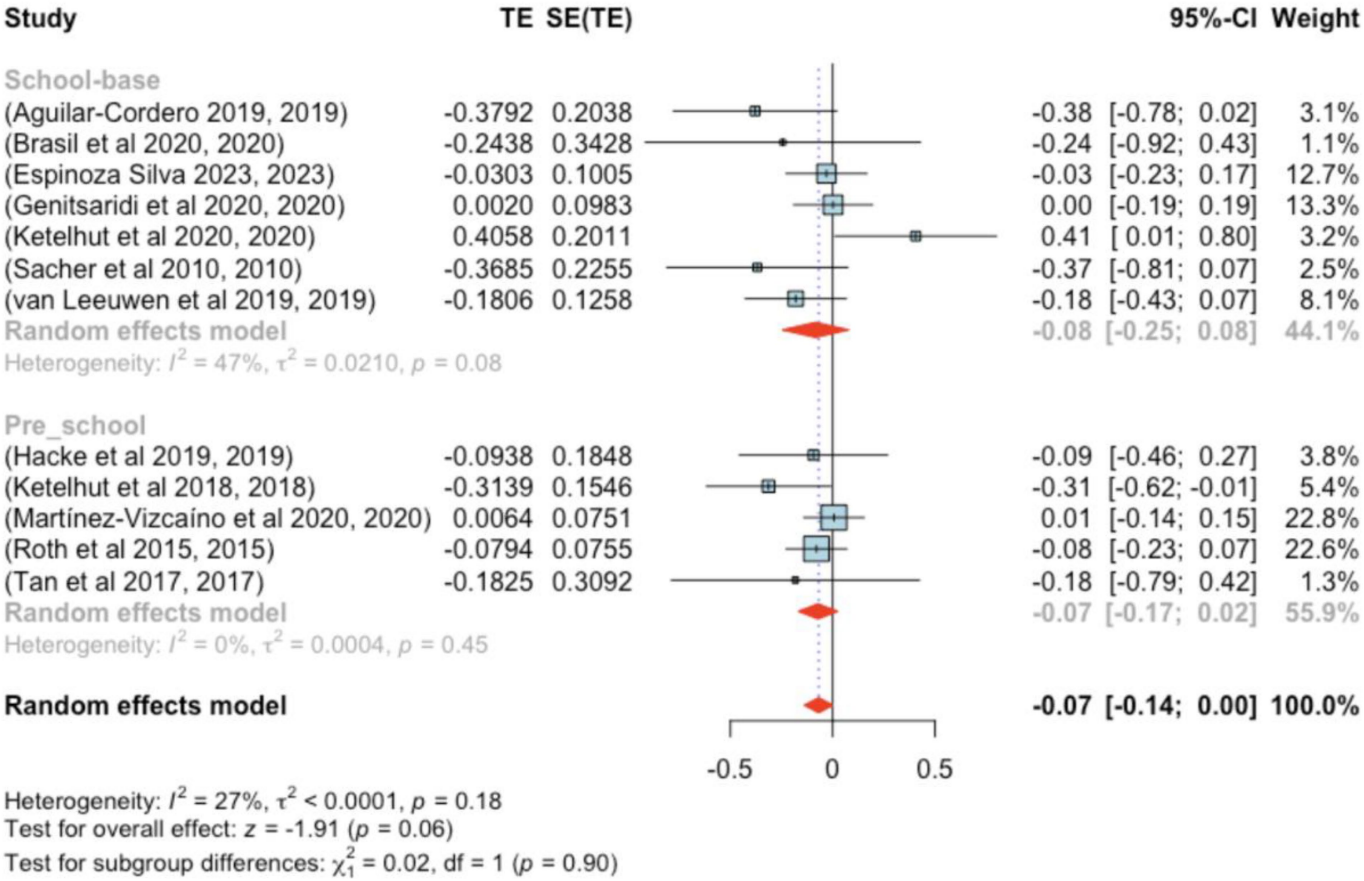
Forest plot showing the observed outcomes and the estimate of the random-effects model.

The analysis encompassed 12 studies (*k* = 12). Standardized mean differences ranged from −0.379–0.406, with 75% being negative. The estimated average standardized mean difference was *μ* = −0.068 (95% CI: −0.139–0.002), which did not significantly differ from zero (*z* = −1.909, *p* = 0.056). Heterogeneity was not significant [Q(11) = 15.062, *p =* 0.180, τ^2^ = 0.000, *I*^2^ = 27.021%]. The 95% prediction interval was −0.139–0.002, again suggesting possible positive outcomes. Subgroup analysis between the pre- and school-age groups was not significant (*p =* 0.90) (Figure 10).

**Figure 11:**
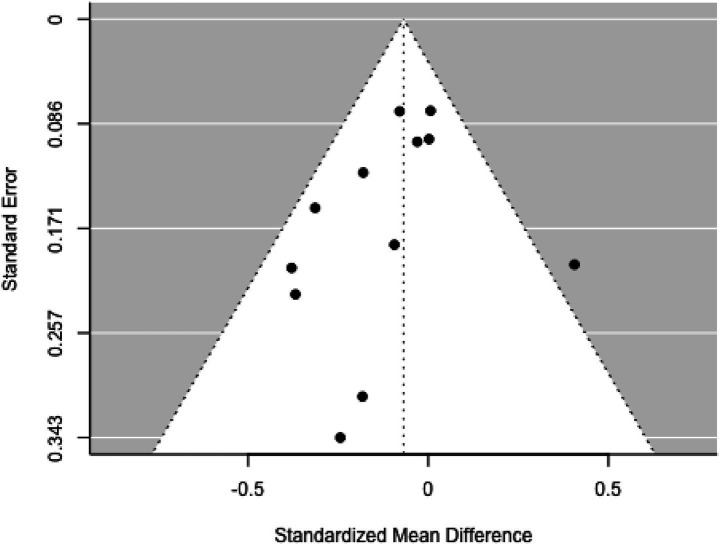
Funnel plot.

No studies had studentized residuals beyond ±2.865, and Cook’s distances did not indicate any overly influential studies. Funnel plot analysis (Figure 11) revealed no significant asymmetry, confirmed by the rank correlation and regression tests (*p =* 0.197 and *p =* 0.175, respectively).

### 3.8 Meta-analysis of SBP outcome

**Figure 12:**
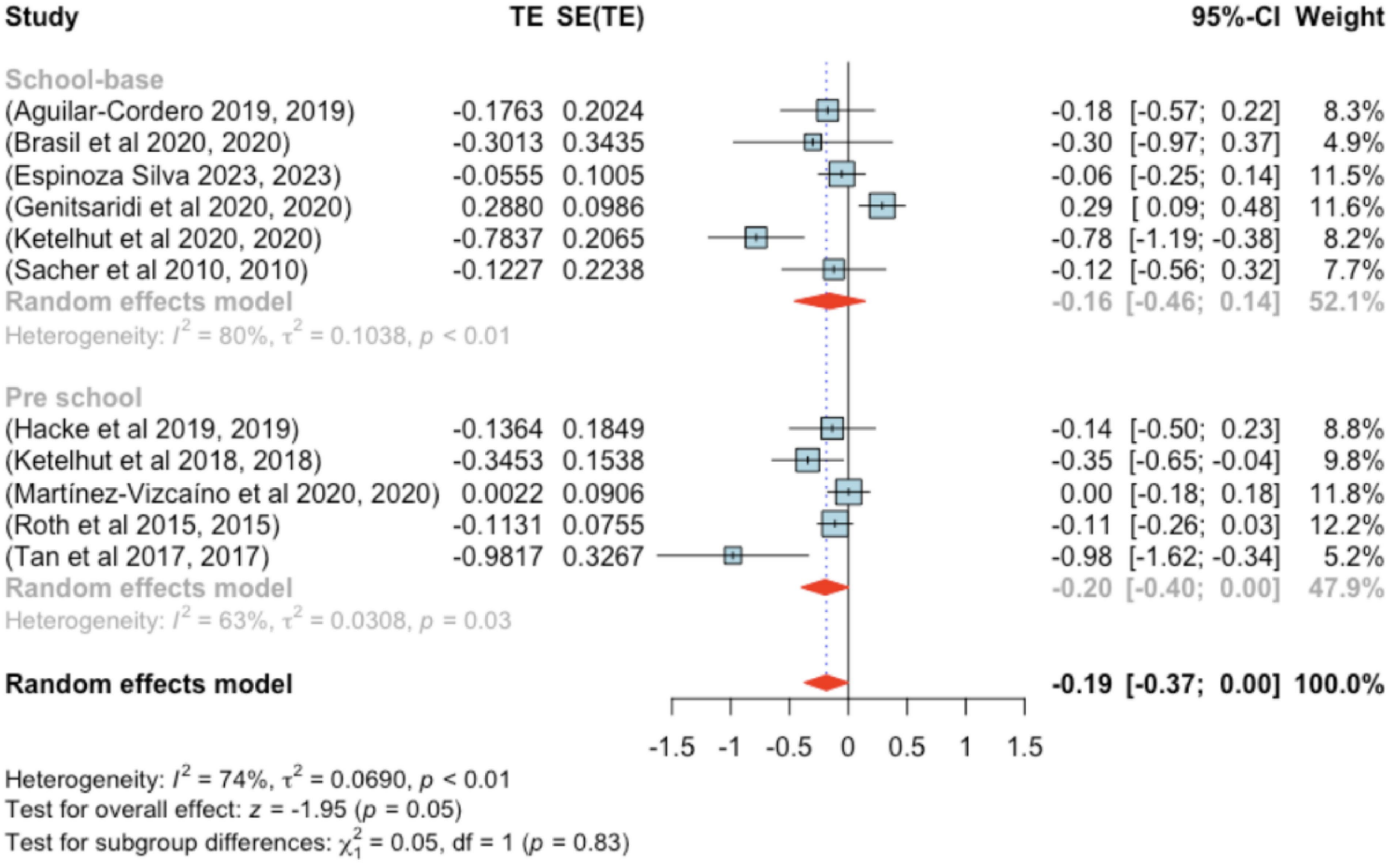
Forest plot showing the observed outcomes and the estimate of the random-effects model.

Eleven studies were included in this analysis. Standardized mean differences ranged from −0.982 to 0.288, with 82% being negative. The estimated average standardized mean difference was *μ* = −0.186 (95% CI: −0.373–0.001). This result did not significantly differ from zero (*z* = −1.951, *p =* 0.051). Heterogeneity was present [Q(10) = 37.979, *p* < 0.001, τ^2^ = 0.069, *I*^2^ = 74.109%]. The 95% prediction interval ranged from −0.734 to 0.361, suggesting the possibility of positive outcomes in some studies. Subgroup analysis between the pre- and school-age groups was not significant (*p =* 0.83) (Figure 12).

**Figure 13:**
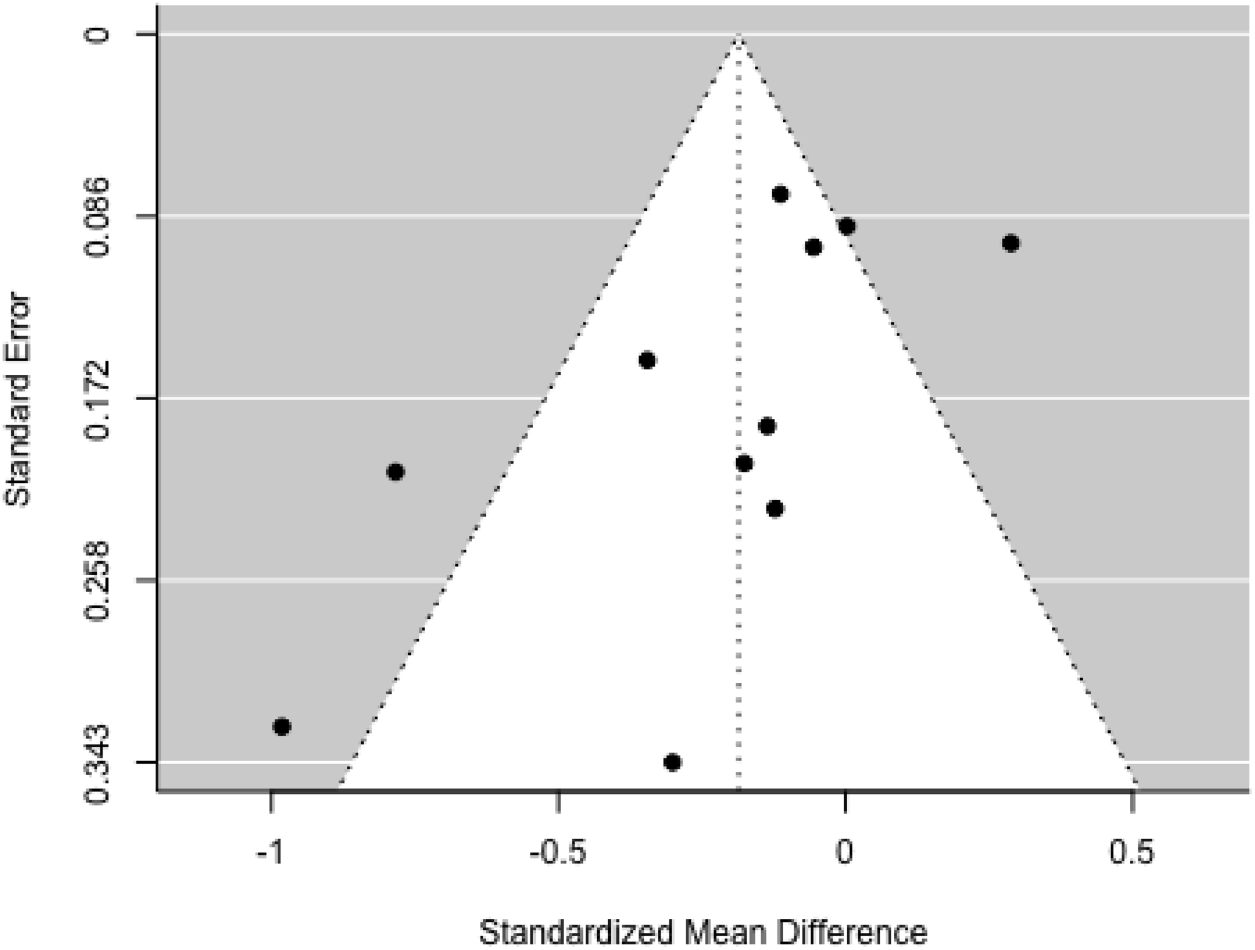
Funnel plot.

None of the studies had studentized residuals exceeding ±2.838, and Cook’s distances indicated no overly influential studies. Funnel plot analysis (Figure 13) indicated significant asymmetry based on the regression test (*p =* 0.007), although the rank correlation test did not indicate bias (*p =* 0.121).

## 4 Discussion

The results of this meta-analysis indicate that physical activity interventions significantly reduce BMI in preschool and school-age children, with similar effect sizes in both age groups. However, effects on other anthropometric indicators (WC, ST, BMI z-score) and physiological parameters (DBP, SBP) tended to be small and mostly not statistically significant. This finding is consistent with previous research (9), which reported a reduction in BMI but found no significant changes in children’s blood pressure.

The reduction in BMI can be explained by biological mechanisms involved in physical activity, such as increased energy expenditure, fat oxidation, and decreased lipogenesis through activation of the AMP-activated protein kinase (AMPK) pathway (54). The largest reductions were observed in three preschool studies (29, 41, 47) and one school-based study (48), all involving obese children. Furthermore, physical activity plays a role in reducing visceral fat and modulating the anti- inflammatory environment by increasing anti-inflammatory interleukins (IL-1ra, IL-6, IL-10), which contribute to improved metabolic profiles.

The benefits of physical activity interventions observed in this study are consistent with those reported in previous research on children and adolescents (55, 56). While the overall impact of these interventions may appear modest, especially given the continued global rise in BMI over the past three decades (“WHO. Childhood overweight and obesity,” 2017), subgroup analysis revealed more noticeable differences in BMI reduction among school-aged children (6–12 years) compared to preschoolers (1–5.99 years). However, this difference was not statistically significant. Moderate changes were also observed in BMI z-score, WC, and ST, though these changes did not reach statistical significance.

Contrasting results have been reported in other studies. Fitzgibbon et al. (29) found significant changes in BMI z-score, while Bocca et al. (25) and Tan et al. (57) observed substantial reductions in WC in preschool-aged children. Similarly, Sacher et al. (44) reported marked improvements in WC, and Tan et al. (48) identified significant changes in ST among school-aged children. These findings align with results from a recent systematic review (9). Furthermore, a longitudinal study found that vigorous physical activity in early childhood is associated with lasting benefits for body composition and physical fitness (58).

The minimal effects on WC, ST, and blood pressure are likely influenced by several factors. First, the majority of participants had normal blood pressure at baseline, limiting the scope for improvement. Second, many interventions were short-term (<12 weeks), even though cardiovascular adaptations require longer time. Third, most studies did not control for other lifestyle factors such as diet and sleep duration, potentially confounding the true effects of physical exercise.

These findings also indicate that program duration and intensity play a significant role. Studies with durations of ≥24 weeks tended to show greater improvements in cardiovascular parameters than shorter programs. For example, Ketelhut et al. (33) reported a significant reduction in DBP after a 96-week program, while a 5-week program (36) showed limited changes.

From a policy perspective, these results underscore the importance of integrating structured physical activity into school curricula and preschool programs. Physical activities that combine aerobic exercise, gross motor skills, and play elements can increase children’s participation while providing long-term health benefits.

However, several limitations of this study should be noted. The high heterogeneity in some analyses (e.g., BMI z-scores with *I*^2^ > 90%) indicates significant variation between studies, both in program design and participant characteristics. Furthermore, most studies were from high- income countries, so generalization to developing country populations should be approached with caution. Underreporting of exercise intensity and the absence of dietary control also limit the interpretation of the results.

## 5 Conclusion

This meta-analysis provides strong evidence that physical activity interventions are effective in reducing BMI in preschool and school-age children, with no significant differences between the two age groups. However, the effects on waist circumference, skinfold thickness, BMI z-score, and blood pressure were relatively small and non-significant.

To maximize health benefits, physical activity programs should be structured, long-term, and moderate to high-intensity, and combined with other lifestyle interventions such as nutrition education. Future research should expand coverage to low- and middle-income countries, report exercise intensity in detail, and control for lifestyle factors that may influence outcomes.

Implementing policies mandating daily physical activity in schools and preschools could be a strategic step in preventing obesity and improving children’s overall health.

## Data Availability

The data analyzed in this study is subject to the following licenses/restrictions: If the journal ask the dataset, we will give it. Requests to access these datasets should be directed to Nina Wang, wqlwnn@gmail.com.
